# Risk factors associated with slide positivity among febrile patients in a conflict zone of north-eastern Myanmar along the China-Myanmar border

**DOI:** 10.1186/1475-2875-12-361

**Published:** 2013-10-10

**Authors:** Nana Li, Daniel M Parker, Zhaoqing Yang, Qi Fan, Guofa Zhou, Guoping Ai, Jianhua Duan, Ming-chieh Lee, Guiyun Yan, Stephen A Matthews, Liwang Cui, Ying Wang

**Affiliations:** 1 Department of Tropical Disease, Institute of Tropical Medicine, Third Military Medical University, Chongqing, China; 2 Department of Parasitology, Kunming Medical College, Kunming, China; 3 Department of Entomology, Pennsylvania State University, 501 ASI Building, University Park, PA, USA; 4 Department of Anthropology, Pennsylvania State University, 409 Carpenter Building, University Park, PA, USA; 5 Dalian Institute of Biotechnology, Dalian, Liaoning Province, China; 6 Program in Public Health, University of California at Irvine, Irvine, CA, USA; 7 Population Research Institute, Pennsylvania State University, 601 Oswald Tower, University Park, PA 16802, USA; 8 Department of Sociology, The Pennsylvania State University, 601 Oswald Tower, University Park, PA 16802, USA

**Keywords:** Plasmodium falciparum, Plasmodium vivax, The Greater Mekong Subregion, Myanmar, China, Border malaria

## Abstract

**Background:**

Malaria within the Greater Mekong sub-region is extremely heterogeneous. While China and Thailand have been relatively successful in controlling malaria, Myanmar continues to see high prevalence. Coupled with the recent emergence of artemisinin-resistant malaria along the Thai-Myanmar border, this makes Myanmar an important focus of malaria within the overall region. However, accurate epidemiological data from Myanmar have been lacking, in part because of ongoing and emerging conflicts between the government and various ethnic groups. Here the results are reported from a risk analysis of malaria slide positivity in a conflict zone along the China-Myanmar border.

**Methods:**

Surveys were conducted in 13 clinics and hospitals around Laiza City, Myanmar between April 2011 and October 2012. Demographic, occupational and educational information, as well as malaria infection history, were collected. Logistic models were used to assess risk factors for slide positivity.

**Results:**

Age patterns in *Plasmodium vivax* infections were younger than those with *Plasmodium falciparum*. Furthermore, males were more likely than females to have falciparum infections. Patients who reported having been infected with malaria during the previous year were much more likely to have a current vivax infection. During the second year of the study, falciparum infections among soldiers increased signficiantly.

**Conclusions:**

These results fill some knowledge gaps with regard to risk factors associated with malaria slide positivity in this conflict region of north-eastern Myanmar. Since epidemiological studies in this region have been rare or non-existent, studies such as the current are crucial for understanding the dynamic nature of malaria in this extremely heterogeneous epidemiological landscape.

## Background

Malaria is an enormous public health threat and a detriment to development in Southeast Asia [1]. While most current malaria control efforts have been geared toward Africa, Southeast Asia remains an important but surprisingly neglected malarious region. Around 70% of the total population of this region is at risk of contracting malaria, with 26% at high risk (i e, areas with a reported incidence of greater than one case per 1,000 population per year). Within Southeast Asia, the Greater Mekong Sub-region (GMS), which is made up of Cambodia, China’s Yunnan Province, Laos, Myanmar, Thailand, and Vietnam, remains an important epicentre of malaria despite improvement in the malaria situation in recent years [2,3]. In particular, the GMS has been a breeding ground for multidrug resistant parasites. In the past*, Plasmodium falciparum* parasites resistant to chloroquine and pyrimethamine have emerged here and spread to Africa [4,5]. Therefore, the recent detection of resistance to the front line treatment artemisinins in the GMS has raised considerable concerns [6,7], meaning that the impact of malaria control efforts here reach far beyond this region. In recognition of this serious threat, the World Health Organization (WHO) has deployed intensified malaria control efforts with the aim of containing artemisinin-resistant parasites [8].

Within the GMS, the malaria distribution is tremendously heterogeneous. While some countries are aiming to eliminate or eradicate malaria in the near future, some are struggling to control malaria. Furthermore, malaria heterogeneity in each GMS country is reflected in the clustering of malaria along international borders. The uneven distribution within a country often requires that limited control resources are targeted towards these hotspots. These efforts in turn rely heavily on accurate knowledge of malaria epidemiology.

Myanmar has the heaviest malaria burden in the GMS and, with its unique geographical location, it plays an important role in regional malaria transmission. In the east it borders Thailand, where decreased sensitivity to artemisinins has recently been detected [9], whereas in the west it borders with India, where the malaria burden is among the heaviest [10]. This suggests that, with its ~200,000 malaria cases/year, Myanmar could serve as an important amplifier for accelerating the spill-over of drug resistance to other regions (such as Africa). Moreover, the introduction of malaria from Myanmar to neighbouring countries, such as China and Thailand, by large, cross-border, migratory human populations hinders malaria elimination efforts in these countries.

Even within Myanmar malaria is distributed unevenly and the most malarious regions border China, Thailand and India. These same sub-regions tend to also have high proportions of ethnic minorities who are frequently at the margins of society and lack adequate health care [11]. High seasonality in the number of cases, as well as large population movements, means that outbreaks occasionally occur in naïve populations, perhaps especially in Myanmar. For example, remote border regions, such as Kachin State, have some of the highest estimated malaria incidence, morbidity and mortality rates in the region [12].

Political instability and military conflicts have likely exacerbated the malaria situation in these regions. Myanmar is extremely diverse ethnically, with around 135 different self-defined ethnic groups living within its borders, and several of these groups have been involved in active military conflicts with the ruling government over the last half century [13]. Populations residing in conflict areas receive little economic, public health, and medical attention and consequently tend to experience adverse health outcomes including malaria [14]. In addition to negative health outcomes, epidemiologic and demographic data from such regions are understandably scarce [15,16]. Most of these areas have been off limits to foreigners and outside organizations and accurate censuses and epidemiological surveys are lacking. For example, Myanmar has not had an official census since 1980s and even this census may not accurately count ethnic groups that were involved in armed conflicts with the government. Such a lack of information about conflict regions means that not only do the populations living within these areas probably experience poor health outcomes; the extent to which they do so is not well understood. Research that documents and analyses the health of these populations is therefore crucial for planning public health efforts and policy.

Over the past two decades, particularly since the launch of the Mekong Malaria Initiative by the WHO, intensified malaria control efforts have greatly reduced malaria mortality and morbidity in a number of GMS countries [3]. These apparent successes have inspired several GMS countries, such as China, to refocus their programme strategies from malaria control to malaria elimination. In Myanmar, malaria incidence appeared to have also been on the decline. However, information about malaria epidemiology in Myanmar is far from complete and there is a great need for an accurate assessment.

Shortly after the present study began in 2011, an armed conflict erupted between the Kachin Independence Army and the Myanmar government, making wide-scale epidemiological sampling and surveillance challenging at best. However, medical staff was maintained at 13 clinics along the China-Myanmar border and demographic data, blood slides, and questionnaires were collected from febrile patients who presented at the clinics. The aim of this study was therefore to identify risk factors associated with slide positivity among febrile clinic attendees in these 13 clinics. Slide positivity studies can subsequently help inform clinical decision making and provide at least some information about the epidemiological situation in the catchment area [17].

## Methods

### Study region and data

**Figure 1:**
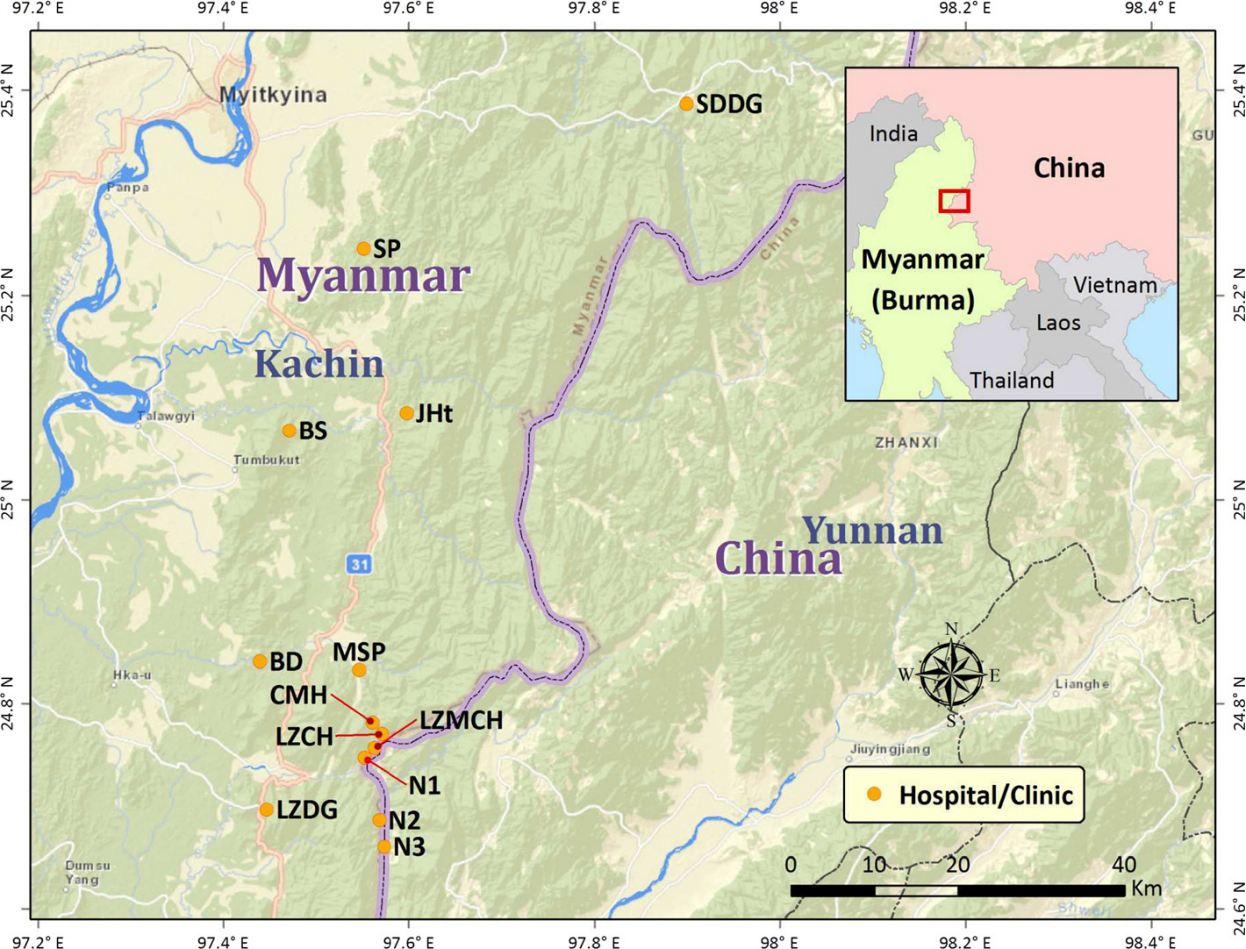
**Map of the study location.** Clinics are marked as yellow dots and purple line denotes the international border. All the clinics involved in the study are located on the border area of Myanmar and China.

The study area included 13 clinics and hospitals in and around Laiza city, Kachin State, Myanmar along the China-Myanmar border (Figure 1). Kachin State is a predominately agricultural state and the vast majority of the State’s estimated 1.2 million inhabitants are ethnic Kachin, also known as Jingpo in China. This study includes patients who presented with fever at health centers in this study area between April 2011 and October 2012. Informed consent was obtained from patients prior to being included in this study and the overwhelming majority of patients gave consent (absolute numbers were not recorded). Consenting patients were then interviewed using a face-to-face survey that included questions concerning demographic attributes, occupation, education, mosquito prevention history, and malaria experience in the previous 12 months. After filling out the questionnaire, a blood smear was made and microscopic examination was carried out in the local clinics. Blood smears were further examined at another nearby field station by two experienced microscopists. Any discrepancies were re-evaluated to obtain a final consensus of the diagnoses.

Patients diagnosed with malaria by microscopy were treated according to the standard protocols of the Health Unlimited organization. Adult patients with *P. vivax* were treated with chloroquine for three days by four tablets as the first dose, two tablets at 24 h and two tablets at 48 h after the first oral administration, and primaquine for eight days by three tablets once per day. *Plasmodium falciparum* positive adult patients were treated with dihydroartemisinin and piperaquine phosphate tablets for three days, with two tablets as the first dose, two tablets 6 h later, two tablets 24 h later, and two tablets 48 h later.

The study protocol was reviewed and approved by institutional review boards from Pennsylvania State University and Kunming Medical University. There is no Kachin institutional review board, however the field team met with representatives of the Kachin Independence Organization prior to beginning the study and received verbal consent to pursue the research.

### Data analysis

The resulting dataset consisted of febrile patients, both with and without malaria infections. The data were therefore divided into three different comparison groups: individuals with *P. falciparum* infections, individuals with *P. vivax* infections, and individuals without malaria infections. In these analyses, comparisons were made between each of these three groups to look for potentially important demographic, occupational, and educational factors as well as malaria infection history (self reported infection within the last 12 months). Multiple logistic regression models were used to test for statistically significant differences between sex, age groups, occupation (Additional files 1, 2, 3 and 4), education (primary education or less versus middle school or higher), and malaria infection history (whether the patient had been infected within the last 12 months) with regard to each of the three aforementioned comparison groups. Questionnaires also asked about bed net usage and whether or not a patient had travelled in the last two weeks. However, both of these variables suffered from poor response levels and data quality problems and were therefore considered potentially be unreliable. They were analysed in Additional file 5, 6, 7 and 8, but left out of the main statistical analyses here.

Subsequent logistic models were used to look for patterns in reported malaria infection history among these same model covariates. The resulting logistic model coefficients were exponentiated in order to present them as odds ratio estimates.

## Results

### Demographic characteristics

Over the study period, between April 2011 and October 2012, a total of 7,089 febrile patients were enrolled and diagnosed for malaria infection by microscopy. The age structure and slide positivity results of enrolled patients are summarized in Table 1. Of the 7,089 enrolled patients, 46% were female, 21% were under the age of five, and 4% reported having a malaria infection in the previous year. Additionally, 24% were farmers, 26% were office workers or students, 34% were houseworkers or non-school children, 3% were listed as “other”, and 13% were soldiers.

**Table 1:** Distribution of slide positive cases and non-cases in the dataset by parasite species and age group

		**Malaria cases**			**Slide positivity (%)**		
**Age group**	**Febrile patients**	**Mixed**	**Pf**	**Pv**	**Mixed**	**Pf**	**Pv**
**0 to 4**	1525	3	16	61	0.20	1.05	4.00
**5 to 14**	1564	20	43	138	1.28	2.75	8.82
**15 to 24**	1408	8	63	115	0.57	4.47	8.17
**25 to 34**	1204	8	42	45	0.66	3.49	3.74
**35 to 44**	670	2	17	22	0.30	2.54	3.28
**45 to 54**	406	3	8	15	0.74	1.97	3.69
**55 to 64**	216	0	3	9	0.00	1.39	4.17
**65 +**	95	1	1	6	1.05	1.05	6.32

Not shown here are four *P. malariae* infections and one *P. ovale* infection.

### Malaria infections as a relatively minor component of febrile illness

Of the 7,089 enrolled patients, only 655 (9.2%) were found to be infected with malaria parasites by microscopy (Table 1). All four major human malaria parasites were present, but the vast majority were *P. vivax* and *P. falciparum* (Table 1). *Plasmodium vivax* is the predominant parasite species and accounted for 71.3% of all malaria cases. Only four *Plasmodium malariae* infections and one *Plasmodium ovale* infection were detected among the patients. A total of 45 patients carried mixed infections by *P. falciparum* and *P. vivax*.

### Temporal trends in malaria cases

**Figure 2:**
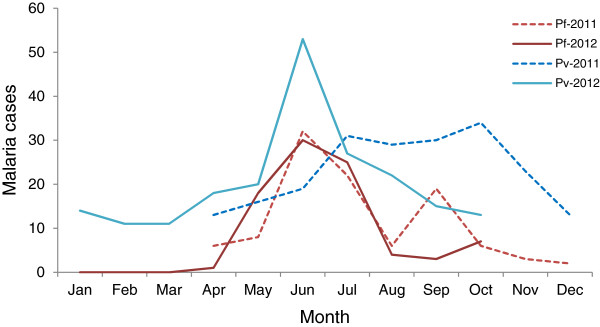
**Malaria cases over time.** Shown are the trends of microscopically confirmed *P. falciparum* and *P. vivax* cases in 2011 and 2012 by month.

Trends in case numbers from these clinics varied greatly over time, but basically followed the rainy season (Figure 2), which typically begins in April. Malaria slide positive case numbers generally peaked in June-July. While the data did not cover a long enough period of time to be conclusive, there appeared to be a double peak at the beginning and at the end of the rainy season (September-October). Compared to 2011, there were declines in both *P. vivax* and *P. falciparum* cases for the period of April-October in 2012.

### Risk factor analysis of malaria infections

There were strong age patterns in slide positivity rates for both *P. vivax* and *P. falciparum* infections (Table 1). Slide positivity peaked for *P. vivax* cases in age group 5 to 14 whereas for *P. falciparum* it peaked in age group 15 to 24 (Additional file 9). Odds ratio estimates from our logistic models indicate that, using the 0 to 4 age group as the comparison group, the 5 to 14 age group has approximately twice the odds (OR: 2.11; CI: 1.33, 3.31) of having a vivax positive slide and the 15 to 24 age group has close to four times the odds (OR: 3.76; CI: 1.74, 8.18) of having a falciparum positive slide (Additional file 9). A comparison of age groups between falciparum and vivax infections indicated that, given that a patient had a malaria positive slide, those in the 25 to 34 age group had about three times the odds (OR: 3.02; CI: 1.19, 7.79) of having a falciparum infection rather than a vivax infection.

The effect of sex was only significant for falciparum infections. Males were more likely than females to be diagnosed with falciparum infection (OR: 1.80; CI: 1.29, 2.54). Interactions between age and sex were also tested. The males in the age groups 15 to 24 and 25 to 44 were significantly more likely to have positive vivax slides, when compared to females of the same age groups. The same interaction term in the falciparum logistic model did not reveal any significant combinations.

There was no effect of education on slide positivity rate. The study population is primarily an agricultural population and farmers were used as the comparison group in the logistic model. There was no significant effect of occupation group with regard to *P. vivax* infections. However, soldiers were less likely than farmers to have *P. falciparum* positive slides (OR: 0.40; CI: 0.15, 0.92). This pattern changed drastically over the study period (Additional file 2 for *P. falciparum*; Additional file 1 shows the same for *P. vivax*), as the aforementioned conflict emerged, and an interaction between study year and occupation revealed a statistically significant increase (OR: 3.62; CI: 1.32, 11.33) in falciparum slide positivity in soldiers during 2012 (Additional files 2 and 9).

### Infection history and slide positivity risk

Febrile patients with a history of malaria infection in the last year were significantly more likely to have a current vivax positive slide. The logistic model indicated that patients with a recent history of infection had about three and a half times the odds (OR: 3.45; CI: 2.42, 4.83) of vivax infection when compared to those with no recent history of malaria infection. There was no statistically significant effect of recent infection history for falciparum infections. However, a comparison of only falciparum and vivax positive patients indicated that those with recent malaria infections had about half the odds (OR: 0.44; CI: 0.22, 0.86) of falciparum infection.

The demographic characteristics of individuals who reported having a history of malaria infection (having been infected in the last year) were further investigated using a logistic model (Additional file 10). This model indicated that individuals between the ages of 15 and 34, males, and those with current malaria positive slides were most likely to have been infected with malaria in the last year. Furthermore, office workers and students, those with greater than a primary school education, houseworkers and non-school children, and those who reported using bed nets were significantly less likely to have had a recent malaria infection. For this model, the occupation and year interaction terms were left out, because the small number of patients reporting previous infections led to a poor model fit.

## Discussion

The original plan for this study region had been to conduct active surveillance, coupled with demographic surveys, in order to investigate risk patterns in malaria infections. However, military fighting began shortly after the field work began and the subsequent dangerous circumstances limited the researchers’ ability to conduct population-wide sampling and surveillance. Instead, medical staff were maintained at 13 different clinics along the border near Laiza, Myanmar and Nabang, China. Since mid-2011 fighting in Kachin state has displaced several hundred thousand people to areas in and around Laiza. These displaced individuals, as well as the villagers who already lived in the area, were covered by the clinics and hospitals involved in this study.

The changing circumstances in the field probably also influenced the epidemiological situation in this area. Human migration has previously been implicated in border malaria within Southeast Asia [18,19]. In this situation, villagers fled from deeper within Kachin State where military fighting was occurring and moved nearer to the China-Myanmar border. Many of these internally displaced people lived or currently live in hastily built, packed, row shelters. Such close quarters and poor conditions may lead to poor health conditions and the easy spread of directly transmitted infectious diseases. Previous researchers have discussed the implications of migration, and forced migration in particular, with regard to various health outcomes [16,20,21]. Because of the dangers inherent in working in a conflict zone, the researchers were unable to accurately document the extent to which the general population grew during this time period.

Regardless of the changing demography, malaria cases continued to exhibit the seasonal transmission that is typical of this region, peaking in May-July. While these time-series data are limited, the data appear to suggest a double peak; a pattern that is typical of malaria in other regions of the GMS, such as western Thailand and China’s Yunnan province [22-24], which mostly reflects the dynamics of the monthly rainfall in this region. Four species of the human parasites were found in this region, but *P. vivax* and *P. falciparum* infections accounted for the majority of malaria cases. Mixed species infections (*P. vivax/P. falciparum*) accounted for approximately 7% of all infections (Table 1). Further, *P. malariae* and *P. ovale*, though detected at very low levels, are often missed by microscopy [25]. Because different parasites need to be treated differently, more sensitive and accurate diagnostic methods are needed for malaria elimination campaigns in this region.

Age-dependent patterns of malaria were observed for both *P. vivax* and *P. falciparum* infections. For vivax infections, slide positivity peaked in children between the ages of 5 and 14 years and in young adults aged 15 – 24 years. The age of peak vivax slide positivity, and the lack of an effect for sex (especially in this age group), are both parsimonious with transmission occurring in or near the village, where children spend the majority of their time. Furthermore, this study showed that individuals with a history of infection had about three and a half times the odds of having a vivax positive slide. Febrile patients with a history of malaria infection had slightly older age profiles (aged 15 through 34) and were disproportionately male. It is possible that these individuals are migrants who are at increased risk of infection; anecdotally, patients who reported having travelled in the previous two weeks were also more likely than others to report previously having been infected. However, we are cautious in these interpretations since patients with reported infection histories only made up approximately 4% of the patients included in this study. It is also possible that these are actually recrudescence rather than true re-infections, however it is impossible to differentiate the two from slides alone. If these are true re-infections, this lends further support to the idea that certain subgroups within the population are at greater risk of infection (in this case, even re-infection).

For falciparum infections, slide positivity peaked between ages 15 and 34, the highest being in the 15 to 24 age group. Males were also at increased risk of falciparum infection, however an interaction between sex and age in the logistic model did not reveal any significant interactions. Regardless, this pattern may be the result of a number of different factors. For example, if falciparum occurs at lower transmission intensity than vivax, it is possible to see an average age of infection that is slightly older in comparison to vivax infections. Conversely, there is evidence that as transmission intensity increases, the age of peak morbidity decreases [26-28]. This pattern is thought to be the result of a variety of complex, interrelated factors, including acquired immunity, maternal immunity, and the annual risk of infection.

At least one study has also suggested that in regions with both vivax and falciparum infections, immunity to vivax may develop more quickly, leading to an older age pattern in symptomatic falciparum when compared to symptomatic vivax [29]. However, the region under study here is characterized by hypoendemicity and generally low transmission and conventional thinking is that immunity should not develop under such conditions.

Another possibility for the difference in age patters is that the main vector of falciparum in this area may be different than the main vector of vivax. It is therefore possible that the two malarias have differing ecologies and that the difference in sex and age is a product of differential exposure by these two demographic variables. Males who are working in different regions or ecosystems, for example in agricultural fields, may be disproportionately exposed to falciparum malaria vectors. The biting behaviours of mosquito vectors and their significance in outdoor transmission in this region require future investigations. Nevertheless, similar occupation-related risks of malaria infection in Southeast Asia has been described previously [18,19,30]. In the nearby region of China’s Yunnan province, imported malaria infections were excessively high among working-age males [29], that has been related to business-related travel histories of patients to malaria-endemic areas of Myanmar. Certainly this is the case in the peak in *P. falciparum* cases among soldiers (who are overwhelmingly young-adult males) during the second year of this study (Additional file 2).

In the logistic model for *P. falciparum* risk factors, soldiers are shown to be significantly less likely to have falciparum infections when compared to farmers. This could be an artefact of the changing demography of this region during the study period. While there are no demographic data to accurately analyse these changes in the population, it is likely that soldiers both moved into the area and that farmers already living in the area were recruited as soldiers over the study period as the conflict unfurled. Laiza, Myanmar actually served as the headquarters for the Kachin Independence Army during this period of time. Soldiers were frequently stationed in the forests and hills surrounding the study location and were, therefore, exposed to different ecological conditions when compared to other study patients. Combined with their lack of reported use of bed nets (Additional file 7), it is perhaps not surprising that soldiers represented a large portion of falciparum slide positive cases during 2012.

There are several important limitations to the present study. Perhaps foremost among these is the lack of detailed information about the catchment population for these clinics. Under the serious and sometimes dangerous circumstances in this conflict zone, the researchers were unable to maintain planned demographic and epidemiologic surveys. Accurate surveys concerning the clinical epidemiology in this region were therefore not conducted and it is not possible to estimate malaria incidence or prevalence in the study site. Furthermore, while the questionnaire did ask febrile patients whether or not they had travelled in the previous two weeks, it was not designed to address migration in a detailed manner. Some related issues are the relatively high rate of non-response to this question, the lack of information about why people did not respond to this question, and the two week period of time would be at or less than the amount of time needed for symptomatic malaria to emerge after the point of infection. These issues make it difficult to interpret analyses that include this covariate. Therefore, this covariate was discarded from the main models and it was left to the reader to decide if analyses including it (in additional files) are valid. Future research will take a more detailed look at migration in this region. Also it is important to note that among all febrile patients who presented at the clinic, only about 10% actually had malaria infections. This means that most of these patients had a health condition, with fever as a symptom, but that only a small fraction of them received treatment from the malaria clinics. While this study is not able to address this problem, the conditions surrounding the conflict in this region also make crowd diseases a potential public health threat and future efforts should be directed at diseases other than malaria alone.

Regardless, this study provides novel information about malaria slide positivity and associated risk factors for a conflict region in Kachin State, along the China-Myanmar border. Economic under-development and poor health infrastructure are probably responsible for the persistent levels of malaria endemicity in this and other similar areas, whereas recent military conflicts further aggravate healthcare delivery in this region. Thus, as a result of these problems, accurate epidemiologic and demographic data from this region are scarce. Such data are important not only for public health efforts in this region but also for the overarching GMS since Myanmar is thought to act as a reservoir for falciparum malaria and potentially as a conduit through which drug resistant malaria can spread [3]. Given the strategic importance of malaria in Myanmar for the GMS, this epidemiological study fills an urgent knowledge gap with regard to both continued malaria control efforts and for understanding the shifting epidemiological dynamics as this region undergoes elevated malaria control efforts.

## Abbreviations

GMS: Greater Mekong Subregion; WHO: World Health Organization.

## Competing interests

The authors declare that they have no competing interests.

## Authors’ contributions

ZY, GZ, ML, GY, LC, and YW conceived and planned the study. NL, ZY, GA, JD, ML, and YW carried out field surveys and microscopic diagnoses. QF and YW confirmed diagnoses in the laboratory. NL, DMP, SAM, QZ GY, and YW, analysed the data. NL, DMP, SAM, LW, and YW wrote the paper. All authors helped in editing and revising the paper and all authors read the final version and approved of its submission.

## Supplementary Material

Additional file 1 ***Plasmodium vivax *** **cases by occupation over time.** Description: Area stacked chart indicating the number of cases of *P. vivax* cases, and the occupational groups to which they are attributed, over the study period. Click here for file

Additional file 2 ***Plasmodium falciparum *** **cases by occupation over time.** Description: Area stacked chart indicating the number of cases of *P. falciparum* cases, and the occupational groups to which they are attributed, over the study period. Click here for file

Additional file 3 **The allocation of malaria cases by occupation groups listed in the original questionnaire.** Description: The second column indicates the way that the original occupational groups were grouped to avoid problems associated with small numbers. Click here for file

Additional file 4 **The distribution of malaria cases by occupation groups used in analyses.** Description: This figure indicates the distribution of malaria cases among the occupation groups that were used in the main analysis (Additional file 6). Click here for file

Additional file 5 **Reported bed net usage and reported travelling within the previous two weeks.** Description: Results from logistic regression analysis of reported bed net use and having travelled in the previous two weeks. Click here for file

Additional file 6 **Logistic model results for risk factors associated with malaria slide positivity (including covariates that were omitted (bed net use and recent travelling)).** Description: The data provided here are the results of the main logistic regression model (shown in Additional file 6) but also including reported bed net usage and having recently travelled as covariates. Click here for file

Additional file 7 **Logistic model showing predictors of reported bed net usage.** Description: The data provided here are the results of an analysis into demographic risk factors associated with a patient reporting having used a bed net within the previous month. Click here for file

Additional file 8 **Logistic model showing predictors of having recently travelled.** Description: The data provided here are the results of an analysis into demographic risk factors associated with a patient reporting having travelled within the previous two weeks. Click here for file

Additional file 9 **Logistic model results for risk factors associated with malaria slide positivity.** Description: The data provided here represent the results of the main statistical analysis of the demographic risk factors associated with slide positivity for *P. vivax* infections and *P. falciparum* infections. Click here for file

Additional file 10 **Logistic model for history of malaria infection.** Description: The data provided here are the results of an analysis into demographic risk factors associated with a patient reporting having had a previous malaria infection within the previous year. Click here for file
