# Supplementary material for: Risk factors associated with slide positivity among febrile patients in a conflict zone of north-eastern Myanmar along the China-Myanmar border
Source: Malar J. 2013 Oct 10;12:361. doi: 10.1186/1475-2875-12-361 (PMC3852943; doi:10.1186/1475-2875-12-361)
Supplement: Additional file 1 — Plasmodium vivax cases by occupation over time. Description: Area stacked chart indicating the number of cases of P. vivax cases, and the occupational groups to which they are attributed, over the study period. [file 1475-2875-12-361-S1.pptx]

## Slide 1
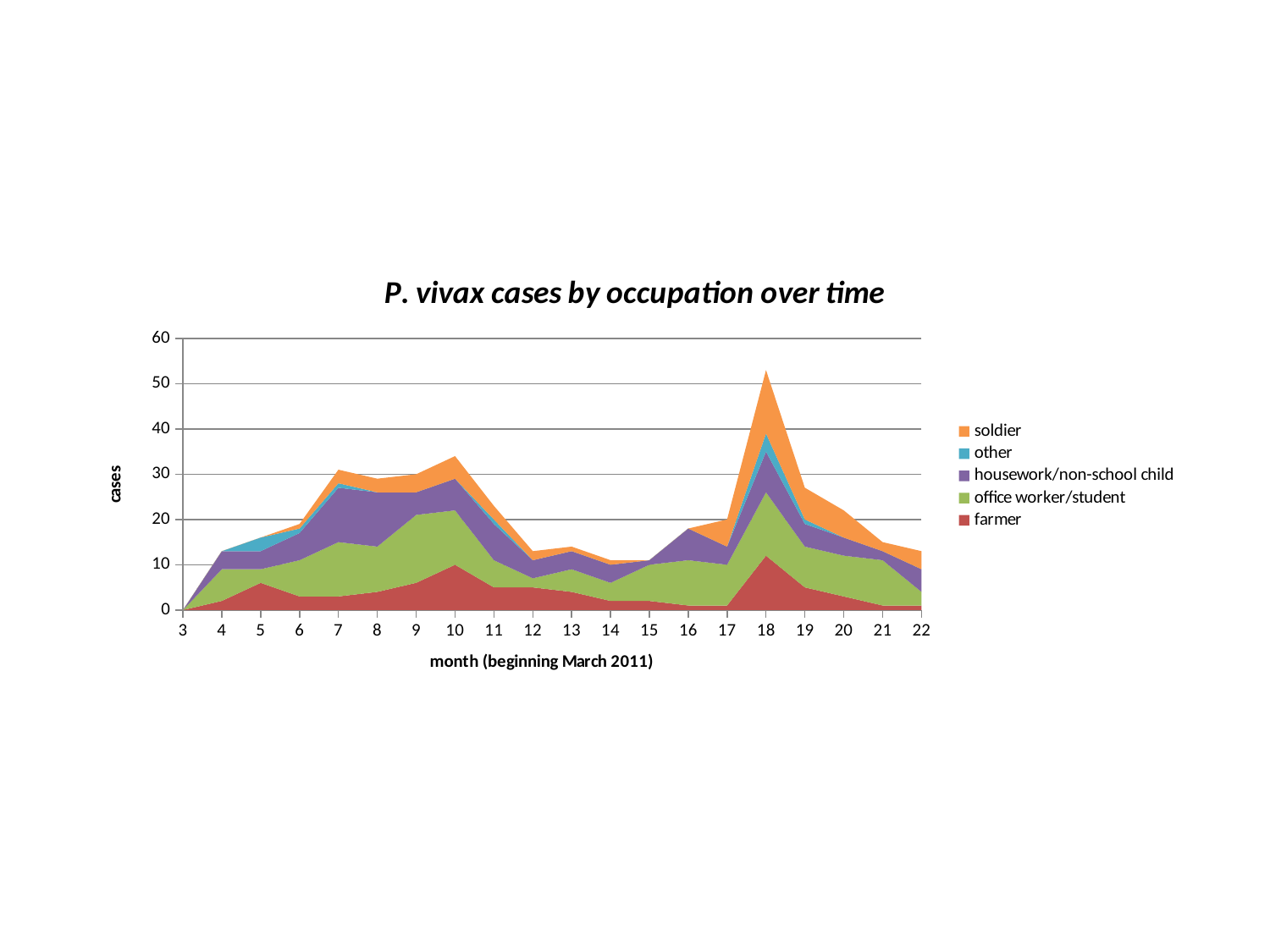

### Chart: P. vivax cases by occupation over time
| Category | farmer | office worker/student | housework/non-school child | other | soldier |
|---|---|---|---|---|---|
| 3 | 0.0 | 0.0 | 0.0 | 0.0 | 0.0 |
| 4 | 2.0 | 7.0 | 4.0 | 0.0 | 0.0 |
| 5 | 6.0 | 3.0 | 4.0 | 3.0 | 0.0 |
| 6 | 3.0 | 8.0 | 6.0 | 1.0 | 1.0 |
| 7 | 3.0 | 12.0 | 12.0 | 1.0 | 3.0 |
| 8 | 4.0 | 10.0 | 12.0 | 0.0 | 3.0 |
| 9 | 6.0 | 15.0 | 5.0 | 0.0 | 4.0 |
| 10 | 10.0 | 12.0 | 7.0 | 0.0 | 5.0 |
| 11 | 5.0 | 6.0 | 8.0 | 1.0 | 3.0 |
| 12 | 5.0 | 2.0 | 4.0 | 0.0 | 2.0 |
| 13 | 4.0 | 5.0 | 4.0 | 0.0 | 1.0 |
| 14 | 2.0 | 4.0 | 4.0 | 0.0 | 1.0 |
| 15 | 2.0 | 8.0 | 1.0 | 0.0 | 0.0 |
| 16 | 1.0 | 10.0 | 7.0 | 0.0 | 0.0 |
| 17 | 1.0 | 9.0 | 4.0 | 0.0 | 6.0 |
| 18 | 12.0 | 14.0 | 9.0 | 4.0 | 14.0 |
| 19 | 5.0 | 9.0 | 5.0 | 1.0 | 7.0 |
| 20 | 3.0 | 9.0 | 4.0 | 0.0 | 6.0 |
| 21 | 1.0 | 10.0 | 2.0 | 0.0 | 2.0 |
| 22 | 1.0 | 3.0 | 5.0 | 0.0 | 4.0 |
