# Supplementary material for: Risk factors associated with slide positivity among febrile patients in a conflict zone of north-eastern Myanmar along the China-Myanmar border
Source: Malar J. 2013 Oct 10;12:361. doi: 10.1186/1475-2875-12-361 (PMC3852943; doi:10.1186/1475-2875-12-361)
Supplement: Additional file 5 — Reported bed net usage and reported travelling within the previous two weeks. Description: Results from logistic regression analysis of reported bed net use and having travelled in the previous two weeks. [file 1475-2875-12-361-S5.docx]

**Additional file 5: Logistic regression of predictors of reported bednet usage and reported travelling within the previous two weeks**

**Reported bednet usage and slide positivity**

Patients who reported using regular, untreated bednets had about a 43% and 59% decrease in the odds of vivax (OR: 0.57; CI: 0.38, 0.89) and falciparum (OR: 0.42; CI: 0.26, 0.71) positive slides respectively. Patients who reported using treated bednets had even lower odds of infection, especially with regard to vivax patients (a 65% decrease in the odds of vivax slide positivity (OR: 0.35; CI: 0.18, 0.66),Additional file 6).

Bednet usage across demographic subgroups within the population was also investigated using a logistic model with a binary outcome (yes/no) indicating whether or not the febrile patient reported using a bednet (Additional file 7). There was no significantly significant difference in reported bednet usage by age group (Supplementary table 5), however reported usage appeared to be lowest among the 15 to 24 and the 25 to 34 age groups (both at approximately 90% (please note that we consider reports for all age groups to be excessively high)). The logistic model indicated that males have about 30% decreased odds (OR: 0.71; CI: 0.55, 0.92) of reporting bednet usage. Reported bednet usage also varied by occupation groups: office workers, students, houseworkers, and non-school children all reported more bednet usage when compared to the comparison group (farmers). For example, those who reported doing housework or were listed as a non-school child had almost three times the odds (OR: 2.85; CI: 1.60, 5.31) of also reporting bednet usage. By contrast, soldiers reported significantly less bednet usage, with about half the odds of reported usage when compared to farmers (OR: 0.50; CI: 0.33, 0.77). Febrile patients with a recent infection history and those with a current infection were also significantly less likely to report bednet usage. A model (not shown here) that included whether or not the patient reported having travelled in the previous two weeks indicated no effect for this covariate. Finally, while overall reported bednet usage increased during the second year (OR: 1.62; CI: 1.04, 2.57), the already low reported usage among soldiers decreased even more (OR: 0.33; CI: 0.18, 0.58).

**Travel within the previous two weeks**

Models that included the dummy variable indicating whether or not the febrile patient had travelled in the previous two weeks were also analyzed. Coefficients from the aforementioned logistic models remained mostly unchanged (Additional file 8). Using non-responses as the comparison group, the model suggests that, with regard to vivax infections, both patients who claimed to have not travelled and those who claimed to have travelled within the previous two weeks had reduced odds of vivax positive slides. Similar results were found with regard to falciparum infections, however the dummy variable for those who had travelled didnot reach statistical significance. Finally, the model only comparing falciparum and vivax infections indicated that, among malaria slide positive patients, those who reported having travelled in the last two weeks were significantly more likely to have falciparum.

The demographic and occupational characteristics of individuals who reported having travelled in the previous two weeks were also analyzed using a logistic model (Supplementary table 6). There were no significant differences with regard to age, however we did find that soldiers, those with greater than a primary education, and those with a recent history of malaria infection were more likely to have travelled in the previous two weeks. (Perhaps most importantly, those with a reported history of infection had about 20 times the odds of also reporting having travelled in the previous two weeks (OR: 20.91, CI: 13.23, 33.12)). Office workers and students were less likely to have reported recent travel.
